# Defining the Molecular Intricacies of Human Papillomavirus-Associated Tonsillar Carcinoma

**DOI:** 10.1177/10732748241310932

**Published:** 2025-05-07

**Authors:** Sneha Sethi

**Affiliations:** 1 Adelaide Dental School, Faculty of Health and Medical Sciences, 50071 The University of Adelaide, Adelaide, SA, Australia

**Keywords:** HPV, genes, biomarkers, tonsillar cancer, molecular

## Abstract

**Background:**

The past decade has shown a sharp incline in the human papillomavirus (HPV) infection associated oropharyngeal carcinoma cases, especially in men younger than 60 years old. Tonsils are one of the key sites, within the oropharyngeal region, which shows malignant changes due to HPV infection, and there is very limited literature to understand the specific dynamics in the tonsillar areas.

**Objective:**

This critical review was undertaken to explore and unravel the bio-molecular interactions and the role of specific proteins associated with HPV infection induced tumorigenesis for the tonsils.

**Design:**

A systematic search of the literature was performed utilising keywords and MeSH terms related to HPV and tonsillar carcinoma in PubMed, Scopus, Embase, and Web of Science without restrictions on dates until July 2023. All studies that reported on molecular biomarkers or genes/genetic proteins in the context of HPV associated tonsillar carcinoma were included in the study.

**Results:**

Preliminary searches revealed a total of 2734 studies of which 23 satisfied the final inclusion criteria and were included. More than 25 proteins and biomarkers were identified, and their role in the malignant process was extracted and compiled. This review also presents a short excerpt on each of the molecules identified to provide a better understanding of the pathogenesis.

**Conclusion:**

Given the rapidly increasing number of cases, there is an urgent need for more focused research on virally induced tonsillar cancers, to develop a better understanding, and for clarity of management and treatment.

## Plain Language Summary

Human Papillomavirus is a common, sexually transmitted infection which effects more than 80% of people at least once in their lifetimes. In most cases it is cleared from the body spontaneously with little or no impact, but some types can persist and induce a cancerous change in the affected organs (cervix, penis, anal, oropharynx, tonsil). To elaborate on any specific site impacted by cancer, the understanding of molecular level events is critical, and information linking different events in the tonsils specifically is sparse, This review is an attempt to primarily collate all the HPV and tonsillar cancer data together, and generate an understanding of how cancer occurs, and different strategies to manage it.

## Introduction

Human papillomavirus (HPV) viruses are a heterogeneous group with circular, double-stranded, non-enveloped DNA and more than 200 genotypes have been isolated. These viruses have an affinity to infect and proliferate in the epithelial and mucous cells.^1^ HPV infection is a sexually transmitted infection,^2^ with women having an 85% chance and men having and 91% chance of acquiring it, at least once in their entire lifetime.^3^ Given the odds, it is not surprising that the global prevalence of this infection has grown considerably over the past few decades.^4^ Global burden of cancer indicates that, HPV infections alone contribute towards more than 80% of anal, more than 30% of oropharyngeal, around 80% of vaginal, and 50% of penile cancers worldwide.^5^

Thirty HPV genotypes are currently clustered as alpha-papillomavirus (‘mucosal HPV types’), characteristic of infecting oro-genital mucosal tracts.1^1^ Although HPV viruses characteristically wash out of the system after a transient phase of usually subclinical asymptomatic infection, certain virus subtypes show persistent behaviours and can initiate malignant changes within the mucosal cells.^6^ This subgroup is termed the “high-risk” subtypes, with currently 14 subtypes recognised under this category.^1^

Tonsils have been identified as the most common site to be infected within the oropharynx.^7^ Traditionally, the incidence of tonsillar cancers was attributed to smoking and alcohol habits, but more recently the cases have been associated with a high-risk HPV persistent infection.^8^ Alarmingly, HPV infection-associated tonsillar cancer cases increased by 30% in a period of 4 years (2005 to 2009), whereas the tonsillar cancer cases not associated with HPV infections did not show similar trends.^9^ Thus, indicating a risk of increasing number of high-risk HPV infections, inducing malignant changes. In 2012, the American Centre for Disease Control and Prevention (CDC) also declared oropharyngeal cancer as the most frequently diagnosed cancer linked with persistent high-risk HPV infections, and the prevalence has now well exceeded the prevalence for cervical cancer.^6^ Although, the rising numbers are worrisome, the good news is that HPV infection-associated tonsillar cancers have a 25% higher 5-year overall survival rate, thus indicating a more favourable prognosis.^10^ The detection of HPV DNA after chemotherapy or radiation therapy has shown to be predictor for risk of recurrence and survival outcomes.^11^ This has clear clinical implications, which helps in determining risks, but data regarding assessment and evaluation of HPV-DNA in tonsillar cancers is sparse, and more research is recommended, exploring this connection.

It has been observed that not all high-risk HPV infections are persistent, and induce cancerous changes, but there are certain molecular interactions which ascertain persistence and progression of cancerous changes. The molecular pathogenesis of HPV infection in cervical cancer has been well researched and documented thus, providing an insight into its probable mechanism in the tonsils as well. The current knowledge based on research is limited and there is lack of dedicated evidence towards tonsils as an HPV affected site. Due to this gap, there is limited understanding of any unique circumstances around designing treatment strategies for hpv-associated tonsillar carcinoma. This review is an attempt to colate the different molecular events, in order to encourage the translation of this knowledge into clinical strategies. Viruses tend to induce cellular interactions and incite certain biomolecules to act in an uncontrolled manner, initiating cellular altercations and promoting tumorigenic environments. The cancer inducing viral - host interactions at a cellular level provide a clear understanding into the progression of the disease as well as encourage the development of targeted molecular therapy. This review aims to provide a comprehensive understanding of the bio-molecular interactions and to elucidate the normal and abnormal functions of proteins involved in HPV infections associated with tonsillar cancers.

## Materials and methods

In order to ensure a comprehensive and complete representation of the current data related to HPV infection-associated tonsillar cancer, a systematic search strategy was employed. After extracting the relevant information, short excerpts on each biomarker and molecule identified, were presented. The possible role in tumorigenesis was highlighted and discussed. This review was inspired by the findings of a previously published systematic review, which evaluated the clinical behaviour of HPV infection-associated tonsillar cancers, as compared to tonsillar cancers not associated with HPV infections.^12^ The findings of the study have been reported following the PRISMA guidelines^13^; however, as the findings collated in the results are narrative in nature, a meta-analysis was not planned for this study. The study has been registered with PROSPERO (CRD42022306602).

Given that no human or animal experiments or data were involved in the curation of this study, no ethical/review board approval was sought.

### Data Sources and Searches

A structured reproducible literature search (Appendix) was performed using keywords and MeSH terms related to human papillomavirus and tonsillar carcinoma. The search was performed in July 2023 in the PubMed (including the operators ‘OR’ and ‘AND’, in combination with subject terms (‘MeSH Terms’) and free text terms (‘Text Word’)) and modified to the Scopus, Embase and Web of Science databases. The academic search engines utilised for completing the search for this study were exhaustive, ensuring capture of all relevant data. Bibliographies of the relevant studies were then manually searched to identify additional relevant studies not captured by the search engines.

### Study Selection

A predefined inclusion criterion was determined for this review (studies specifically evaluating HPV-associated tonsillar carcinoma). No restrictions on study design (cross-sectional, cohort and case-control, retrospective, case reports, case studies, and interventional studies) were imposed, excluding studies presenting secondary evidence, like systematic reviews. Titles and abstracts of all the identified studies were retrieved and analysed against the inclusion and exclusion criteria. All of the studies deemed fit to be included in the full-text review stage were then retrieved and evaluated. Excluded studies included duplicates, studies not including HPV infection-associated tonsillar cancer, or had no full text available.

### Data Extraction and Quality Assessment

Data extraction from the final list of relevant studies was performed and collated to a Microsoft Excel document. Quality assessment of the shortlisted papers was done using the Joanna Briggs Institute (JBI) appraisal tools,1^14^ which is essentially a series on nine questions assessed by two independent reviewers under ‘Yes’, ‘No’ or ‘Unclear’ categories. More information on the quality assessment of included papers has been published elsewhere.^12^ The extracted and recorded data included: characteristics of the studies like country and year of publication, study design, biomarker studied, inference of study exploring the potential role of the particular protein in HPV-associated tonsillar carcinoma and the main findings were further explored.

## Results

### Study Characteristics

**Figure 1.**
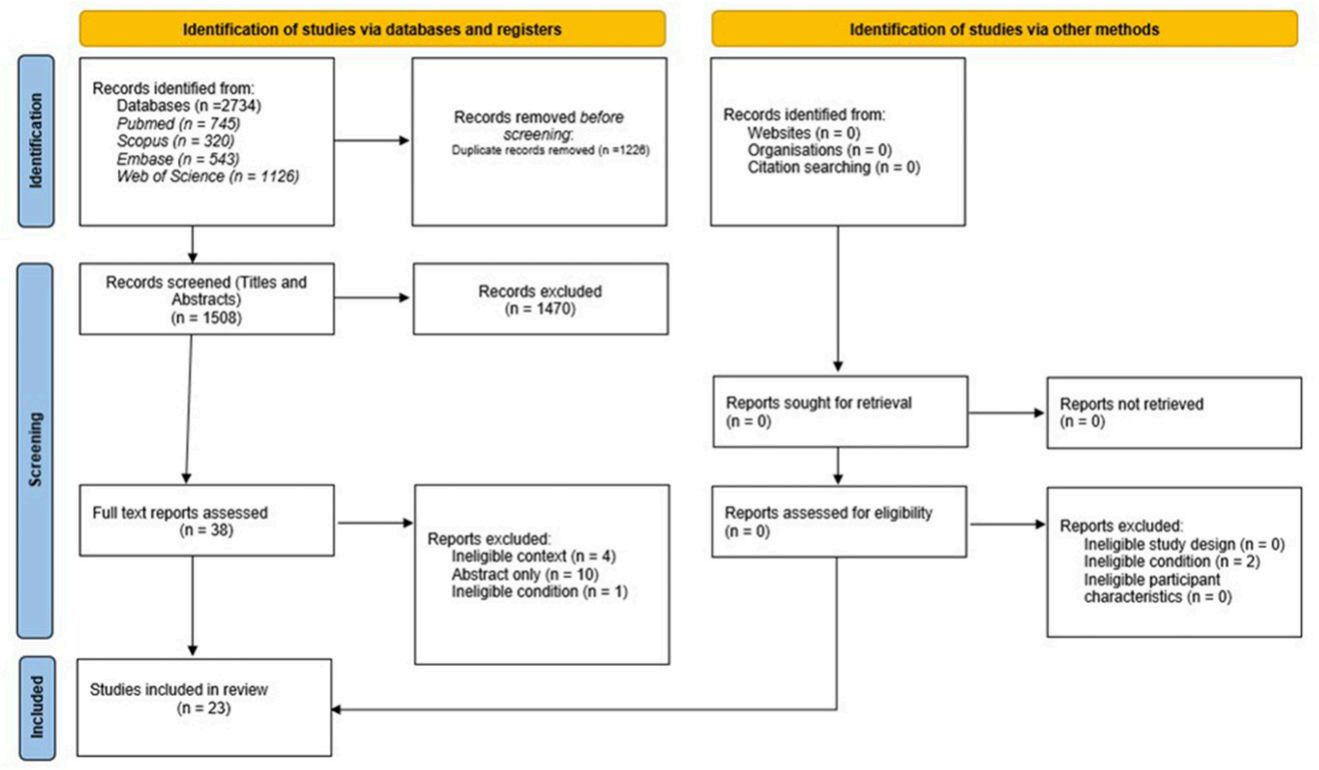
PRISMA 2020 flow diagram for this systematic review which included searches of databases, registers and other sources.

A total of 2734 studies were identified and transferred to the reference managing software, EndNote (X9.3.3). After removal of duplicates, titles and abstracts of 1508 studies were screened. Adhering to the inclusion and exclusion criteria, a total of 38 studies proceeded to the full-text review stage. After reviewing the full texts of the retrieved studies, 23 studies^15–37^ were deemed eligible to be included in the final review (Figure 1).

Geographically, these included studies were from Germany,^15,32,33,35^ Sweden,^16,17,24,28,31,34,37^ USA (United States of America),^18,21^ Netherlands,^19,36^ Republic of Korea,^20,23,27^ Japan,^22,29^ Czech Republic^25,30^ and Italy.^26^ These details and further characteristics of these studies are listed in Table 1.

**Table 1.** Summary of the Characteristics of Studies^15–37^.

Author, Year	Country	Technique	Proteins/Genes/Bio-Markers Studies
Andl T et al (1998)	Germany	IHC^a^, ISH^a^, PCR^a^, MSA^a^, WB^a^	pRb^a^; Cyclin D1
Bersani C, et al (2017)	Sweden	PCR^a^	miR^a^-155, 185, 193b; PIK3CA^a^
Bersani C, et al (2018)	Sweden	PCR^a^	FGFR3^a^; p53
Portugal LG, et al (1997)	USA^a^	PCR^a^	p53
Snijders PJ et al (1992)	Netherlands	PCR^a^, SBH^a^	E6, E7
Snijders PJ et al (1994)	Netherlands	PCR^a^, ISH^a^, IHC^a^	p53
Chun SH, et al (2015)	Republic of Korea	TMA^a^, ISH^a^, IHC^a^, PCR^a^	PTEN^a^
Fury MG, et al (2011)	USA^a^	TMA^a^, IHC^a^	Phosphorylated S6 and phosphorylated eIF4E
Kondoh A, et al (2011)	Japan	IHC^a^, PCR^a^, ISH^a^	Caludin-1,7 and Tricellulin
Lee SH, et al (2014)	Republic of Korea	TMA^a^, PCR^a^, FISH^a^	Syndecan1
Lindquist D, et al (2007)	Sweden	PCR^a^	E6, E7
Salakova M, et al (2018)	Czech Republic	PCR^a^, IHC^a^	E6
Morbini P, et al (2015)	Italy	IHC^a^	Cytokeratin 7, AGR^a^-2, CD-63, MMP^a^-7
Na II, et al (2007)	Republic of Korea	PCR^a^	EFGR^a^
Romanitan M, et al (2013)	Sweden	IHC^a^	EFGR^a^
Näsman A, et al (2013)	Sweden	IHC^a^	Classical HLA^a^-A, B, C
Pham HT, et al (2020)	Japan	IHC^a^	Semaphorin 3A
Pokryvkova B, et al (2020)	Czech Republic	PCR^a^	E2
Ramqvist T, et al (2015)	Sweden	PCR^a^	E2
Quabius, ES, et al (2015)	Germany	PCR^a^, IHC^a^	SLPI^a^
Stenner M, et al (2011)	Germany	IHC^a^	E-cadherin, β-catenin
Tertipis N, et al (2015)	Sweden	IHC^a^	LMP7
Weiss D, et al (2012)	Germany	IHC^a^	Cyclin A1

^a^USA – United States of America, IHC - Immunohistochemistry, ISH – in-situ hybridization, PCR – Polymerase chain reaction, MMA – Microsatellite marker analysis, WB – Western blotting, SBH – Southern Blotting Hybridization, TMA – Tissue microarray, FISH – Fluorescence in situ hybridization, pRb- Retinoblastoma tumour suppressor protein, miR - microRNA, FGFR3 - Fibroblast growth factor receptor 3, PIK3CA - Phosphatidylinositol-4,5-Bisphosphate 3-Kinase Catalytic Subunit Alpha, PTEN - Phosphatase and tensin homolog, AGR – Anterior Gradient, CD - Cluster Differentiation, MMP- Matrix metalloproteinase,^38^ EFGR – Epithelial Factor Growth Receptor, HLA – Human Leucocyte Antigen, SLPI - Synaptotagmin like protein, LMP7 - Low mass protein-7.

Most of the studies used the polymerase chain reaction (PCR) technique,^15–20,22–25,27,30–32,36^ and the other popular choices were immunohistochemistry (IHC),^15,19–22,25,26,28,29,32–35,37^ in-situ hybridization (ISH),^15,19,20,22^ tissue micro-assay analysis (TMA),^20,23^ microsatellite marker analysis (MSA),^15^ western and southern blotting techniques.^15,36^

The role of the different proteins identified in the review are tabulated in Table 2.

**Table 2.** Role of Different Proteins/Genes and Factors in Tonsillar Tumorigenesis Specifically Associated With HPV Infections.

Gene/protein	Role of Gene/Protein in HPV-Positive Tonsillar Cancer	Outcomes
Retinoblastoma tumour suppressor protein (pRb)	Associated with metastasis and tumour invasion.^15^	Unfavorable
miR-155	Positive predictor of survival^16^	Favorable
miR-185	Associated with decreased survival^16^	Unfavorable
FGFR3 (Fibroblast growth factor receptor 3)	Increased expression indicated a worse prognosis for tonsillar cancers^17^	Unfavorable
P53	P53 has not been shown to be a definite indicator of prognosis for HPV infection-associated tonsillar cancers^18,19^	Not definite
PTEN	PTEN nuclear expression was observed in HPV-positive tonsillar cancers, with a favorable survival outcome^20^	Favorable
Phosphorylated S6 and eIF4E	p16 positive tonsillar carcinoma is characterized by the expression of phosphorylated eIF4E^21^	Unfavorable
Claudin-1 and 7	Claudin-1, claudin-7, and tricellulin expressions are associated with tumor growth, invasion and progression in the tonsils, although no specific link to HPV infection has been identified so far.^22^	Unfavorable
SDC1	SDC1 expression was associated with nodal spread tumour resection margins. Positive expression with this marker was associated with unfavorable prognostic outcomes for tonsillar carcinomas.^23^	Unfavorable
E7	E6/E7 expressions have been consistently positive for all HPV-associated tonsillar cases, thus suggesting a key role the tumorigenic process. The E6/E7 transcripts in tonsillar carcinomas could be isolated from integrated as well as episomal DNA.^20,25,36^	Unfavorable
Cytokeratin (CK) 7	It has been postulated that the expression of squamous cell junction markers within the tonsillar crypt cells, could be the potential targets for HPV. The cells might be related to the embryological development of tonsillar structures; their partial could help in developing or identifying specific markers for these tonsillar embryonic cells for identifying HPV infection and malignant transformation. CD-63, CK-7, AGR-2, and MMP-7 could be some of the few markers which could be used to help in estimating the prognosis of HPV infection-associated tonsillar carcinomas^26^	Unfavorable
Anterior Gradient (AGR) 2		Unfavorable
Cluster Differentiation (CD) 63		Unfavorable
Matrix metalloproteinase (MMP) 7		Unfavorable
EFGR (Epithelial factor growth receptor)	The research with EGFR expressions has not indicated any indication of clinical prognosis with tonsillar cancers.^27,37^	Indefinite
Classical HLA-A, B, C (Human leucocyte antigen)	The expression of HLA-A, B, C, irrespective of HPV status may serve as a useful biomarker for prognosis for tonsillar cancers^28^	Unfavorable
Semaphorin 3A (SEMA3A)	The expression of SEMA3A, irrespective of HPV status, is a prognostic marker for survival and has been shown to be associated with angiogenesis in oropharyngeal cancers^29^	Unfavorable
E2	Either negative or no expression of E2 markers was found in HPV-associated tonsillar cancers.^30,31^	Favorable
SLPI (Synaptotagmin like proteins)	Increased SLPI expression has indicated protection against HPV infection of tonsillar and non-tonsillar carcinoma.^32^	Favorable
E-cadherin	The downregulation of E-cadherin expression is involved in tumor progression of tonsillar carcinomas.^33^	Favorable
β-catenin	The up-regulation of nuclear β-catenin expression involved in tumour progression of tonsillar carcinomas.^33^	Unfavorable
LMP7 (Low mass protein 7)	Low LMP7 expression is correlated to better clinical outcomes and has prognostic significance.^34^	Unfavorable
Cyclin A1	Cyclin A1 is an important cell cycle regulator with age-related increased expression in tonsils of children. Exact role in tonsillar cancer development is uncertain, but could be significant.^35^	Indefinite

## Discussion and Review

More than 25 biomarkers were identified through this review (Table 2), and the following section presents a short excerpt on each. The majority of the papers included in the review are from Europe, and a few were from America, Japan and Korea. Given that the prevalence of HPV-associated cancers is higher in lower socioeconomic countries, this paper also highlights the need of more representative data and studies to develop a deeper clearer understanding of the same.

**Figure 2.**
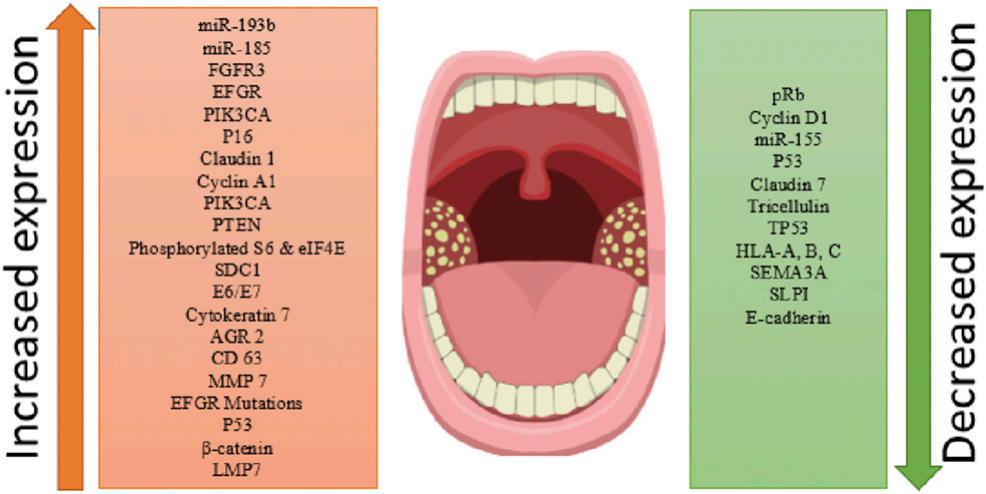
Expression of proteins/genes and factors in tonsillar tumorigenesis specifically associated with HPV infections.

The role of each biomarker in prognosis, tumour formation and metastasis, in cancers of other sites as well as specifically, HPV infection-associated tonsillar cancers is discussed. This overview of the different biomarkers identified in HPV-associated tonsillar cancers can help guide molecular pathways specific to the tonsillar cancers (Figure 2). This will also help in enhancing an understanding of the molecular level events in theses lesions, which could impact potential screening, diagnosis and treatment strategies. Although clinical implications cannot be directly suggested at this stage, but it definitely has the potential to impact clinical management and deliver translatable outcomes for patients diagnosed with HPV associated tonsillar cancer.

### Retinoblastoma Tumour Suppressor Protein (pRb)

First identified in 1971^39^ as a key gene (located on chromosome 13) involved in hereditary retinoblastoma,^40^ pRb plays a significant role in regulating pathways associated with cell cycle,^41^ cell growth,^42^ inflammation,^43^ differentiation,^43^ replication, renewal, turnover and death.^40,43–48^ It has been speculated that in addition to cellular regulation, pRb is also involved in processes impacting tumour growth and extra-cellular interactions like genomic instability and cellular de-differentiation.^43^ The distinct role of pRb as a tumour suppressor protein can be explained by its recognised role in key processes involved in carcinogenesis like proliferating in unsuitable environments and the tendency to metastasize. Another important aspect of pRb associated with tumour initiation and growth, cytokine regulation and research links pRb inactivation with increased secretion of pro-inflammatory cytokines like IL-6,^43^ thus establishing an inflammatory tumour microenvironment. The role of pRb has been reported in most of the cancers like prostrate,^49,50^ breast,^51,52^ small-lung carcinoma,^53,54^ cervix^55,56^ etc.

### Cyclins (D1 and A1)

Cyclins are critical proteins, first discovered in 1982, which facilitate the cell cycle and the processes involved in the cell cycle, by activating enzymes.^57,58^ The cyclin family consists of cyclins (A, B, D, E, F) and cyclin-dependent kinases (CDK1, 2, 4, 6), all of which have distinct functions in the cell cycle and dysregulation of these proteins have been associated with tumorigenic processes.^59,60^

### Fibroblast Growth Factor Receptor 3 (FGFR3)

Located on chromosome 4,^61^ the Fibroblast Growth Factor Receptor gene is responsible for coding this protein. FGFR3 is intricately related to tyrosine kinases. Tyrosine kinases are the specific enzymes within cells that orchestrate the transfer of a phosphate group from an ATP (Adenosine triphosphate) molecule to a protein with tyrosine residues, thus thanatophoric dysplasia, achondroplasia, and hypochondroplasia

### p53

p53 is a tumour suppressor gene that was first identified in 1979 on chromosome 17.^62^ It is critically involved in cell cycle regulation with either DNA reparation or apoptosis induction. Replication of damaged DNA involves p53 to be expressed and produced in the nucleus of the cell. Here, it undergoes chemical modifications^63^ – phosphorylation and acetylation – allowing it to enter its functionally active form to transactivate target genes. The genes targeted are contingent on the extent of DNA damage, as the cell could undergo apoptosis or repair the DNA with the cell cycle being arrested at the G1/S checkpoint.^64^ In the event of severe DNA damage, p53 activates pro-apoptotic genes of the Bcl-2 family,^65^ including Puma and Noxa. Alternatively, in the latter case with p53-mediated cell cycle arrest, p21 undergoes transactivation to repair DNA before the cell returns to the normal cell cycle.^66^ Dysfunctional p53 contributes approximately 38% – 50% of human cancers^67^ with the majority of dysfunctionality being resultant of genomic mutations that prevent appropriate protein conformation.^68^ Somatic p53 mutations are associated with various aggressive carcinomas^69^ as they transactivate oncogenic and growth-promoting genes.^70^

### PIK3CA - Phosphatidylinositol-4,5-Bisphosphate 3-Kinase Catalytic Subunit Alpha

Phosphatidylinositol-4,5-Bisphosphate 3-Kinase Catalytic Subunit Alpha (PIK3CA) is a gene encoding p110 alpha (p110α) which is a catalytic subunit of phosphatidylinositol 3-kinase (PI3K).^71^ PI3K is a critical enzyme mediating the progression of the cell cycle and growth by activating various downstream factors, including PDK1.^72^ Cancer-specific mutations on PIK3CA were discovered in 2004^73^ on chromosome 3,^71^ with alterations increasing PI3K signalling, which in turn promotes growth factor independent development.^71^ In patients testing HPV-positive and having head and neck squamous cell carcinomas, the genetic mutation was seen in 56%, whilst 34%^74^ of HPV-negative patients, had the carcinoma and the mutation.

### PTEN

Phosphatase and tensin homolog (PTEN) is a tumour suppressor enzyme that dimerizes^75^ with another PTEN to allow it to bind to the cell membrane, where it dephosphorylates other enzymes, including PI3K. PTEN further aims to promote DNA repair and assist with chromosome stability.^76^ Discovered in 1997^77^ on chromosome 10,^78^ loss of the PTEN protein is more frequently associated with cancer, than PTEN gene mutations which are seen in approximately 13.5% of all human cancers.^79^ With the loss of the negative regulator for PI3K, the PI3K pathway^80^ is activated with cellular proliferation and survival resulting. Mutated PTEN has a reduced ability to repair damaged DNA and maintain chromosomal stability, hence, a poorer prognosis of cancer is seen from higher penetrance. This is also an outcome of PTEN deficiency as DNA replication and mitotic spindle formation are hindered.^81^ Genetic alterations in PTEN are seen in greater frequencies amongst HPV-positive patients with oral squamous cell carcinomas, in comparison to patients testing HPV-negative.^82,83^ Whilst somatic PTEN mutations are seen in various cancers, there is greater emphasis on endometrial cancers and glioblastomas.^84^

### Phosphorylated S6 and Phosphorylated eIF4E

Phosphorylated S6 (p-S6) and Phosphorylated Eukaryotic Translation Factor Initiation Factor 4E (p-eIF4E) are involved in specific mRNA translation during protein synthesis,^85^ with phosphorylation of these individual proteins being heavily controlled. Phosphorylation is mitigated by downstream effects of the mammalian target of the rapamycin (mTOR) pathway with dysregulation being apparent in various cancers.^86^ p-S6 and p-eIF4E were discovered in 1974^87^ and 1976,^88^ respectively, with elevated levels of either protein being indicative of uncontrolled cell growth^87^ from increased protein synthesis. Enhanced p-eIF4E levels further suppress apoptosis with anti-apoptotic and pro-proliferative mRNAs^89^ being translated.

### Claudin (−1, −7)

Located across 12 chromosomes by over 17 genes,^90^ the claudin family consists of over 24 membrane protein members, each playing a key role in tight junctions. With tight junctions mitigating epithelial cell polarity, loss of tight junctions is closely associated with metastatic potential.^91^ An imbalance in claudin (−1, −7) is seen in increased tumorigenicity of breast tissue. Down-regulation of claudin-1 expression in breast epithelial cells has been seen to have neoplastic effects^92^ with aggressive characteristics. This is similarly seen with claudin-7, as increased cellular discohesion is consistent with high-grade lesions.^93^ Hence, tumour behavior has been seen to be heavily contingent on claudin (−1, −7) expression, with histological grade, invasiveness, and metastatic potential being determined. Tight junctions are further disrupted by HPV,^94^ however, the association between HPV and the claudin members is limited since HPV is seen to instead degrade^95^ MAGI-1.

### Tricellulin

Analogous to its name, tricellulin is a protein found at junctions of three cells in the body, also called MARVELD2.^96^ Intercellular junctional complexes include tight junctions, adherens junctions, and gap junctions, they intercede adhesion between connecting endothelial and epithelial cells.^97^ Due to an epithelial barrier nature, it has been speculated that altered tricellulin expression is associated with advancing cancers, cellular invasion, and metastasis.^98^ Research indicates the impact of altered tricellulin on NF-Κβ pathways and the epithelia-mesenchymal transformation pathways.^98–100^ Studies have reported an altered expression of tricellulin and tight junction proteins in colorectal cancers,^98^ pancreatic cancers,^101^ hepatocellular carcinomas,^102^ and endometrial cancer.^103^ Kondoh et al,^22^ investigated the role of claudin-1,7 and tricellulin tight junction proteins in tonsillar cancers, and although there was some loss of expression for tricellulin junctional proteins in tonsillar squamous cell carcinomas, there was no loss of expression in the cases associated with HPV infections. This study states that HPV infections would not have an impact on the tight junctional proteins, and suggested alternative mechanisms of tumour invasion and metastasis.^22^

### Syndecan 1

Belonging to the four-member syndecan proteoglycan family, this protein is an integral membrane protein with a central core protein with multiple glycosaminoglycans (both chondroitin sulfate and heparin sulfate) side chains.^104^ Due to the involvement of syndecan-1 in cellular processes like proliferation, migration and other cell-cell matrix interactions, it positions itself as an important protein during tumour growth and invasion.^105^ The expression of Syndecan-1 in each tumour type is contextual and its relevance depends on the cell type and its significance. Szatmari T et al,^105^ presents an explorative review of the prognostic role of syndecan-1 with relation to the cellular localisation in different organs. Lee SH et al,^23^ studied the prognostic significance of syndecan-1 in tonsillar cancers and found that in cases with SDC1 positivity, the outcome was unfavourable.

### Cytokeratin (CK) 7

Epithelial cells are structurally comprised of cell organs like mitochondria, ribosomes, Golgi bodies and, cytoskeleton proteins which constitute the structure and function of the cytoskeleton of the cell.^106^ There is an approximately 20 different cytoskeletal proteins, the expression of which is retained by the tumour cells. Cytoskeletal markers are routinely used to diagnose tumours of unknown origin or poorly differentiated cells.^107,108^ Expression of CK-7 is strong in epithelial-derived tumours like colorectal cancers,^109–112^ ovarian cancers^113,114^ and cervical cancers.^115^ Research with specifically HPV-associated tumours has revealed that CK-7 has a role in viral replication, thus promoting high-risk HPV-associated tumorigenesis.^116^

### Anterior Gradient (AGR) 2

Belonging to the protein disulfide isomerase (PD 1) family, the anterior gradient 2 (AGR2) is an estrogen-responsive developmentally regulated gene.^117^ First recognised in breast cancer cells as an estrogen receptor target,^118^ but now also observed as overexpressed in other tumours like ovarian,^119^ pancreatic,^120^ and colorectal cancers.^121^

### Cluster Differentiation (CD) 63

Encoded by the CD-63 gene, this protein is associated with intracellular vesicles inducing functions on the cell surface like cellular signalling for growth, proliferation and motility.^122,123^ Platelet activation is one of its recognised roles,^124^ as well as involvement in abnormal cellular growth leading to tumour formation.^125,126^ The exact role of CD-63 in tumorigenesis is a bit controversial with some studies reporting a negative relationship and a few reporting a positive relationship. A systematic review exploring the prognostic value of CD-63 in different tumours was published in 2018, by Koh et al,^127^ which reported an inverse relationship of CD-63 expression with cancers of the ovaries, breasts, colon and lungs.^123,128–131^ However it was found that CD-63 although not overly expressed in the tumor cells helped in the increased production of proteins which promote metastasis and invasion, thus showing higher levels in the plasma as compared to the tumor tissue itself.^132,133^

### Semaphorin 3A (SEMA3A)

Semaphorin 3A is encoded by SEMA3A genes and belongs to the Semaphorin protein family, which are associated with nervous system development. These proteins are also identified as tumour suppressors, many cells possess Semaphorin receptors on their surface and inhibit cellular division and spread.^134^ The connection to axons of neurons explains its expression in Alzheimer’s disease,^135^ systemic lupus erythematosus,^134^ schizophrenia,^135^ scar tissues, rheumatoid arthritis,^134^ and spinal cord injuries.^136^ Additionally, SEMA3A is linked to multiple roles i.e., immune regulation, vascularization and angiogenesis, bone remodelling, organ formation, embryonic development, chemo repellent and oncogenesis^137^

### Synaptotagmin Like Proteins

One of the primary functions of the cells in our body is to secrete molecules (hormones, lipids, neurotransmitters, etc.) in response to an external stimulus. These molecules are secreted in the form of membrane-bound vesicles by a process called exocytosis. This process includes a step that would show the contact between the membrane of the vesicle and the cell membrane before being secreted from the cell. This contact is mediated by the formation of phospholipid domains called C-2 domains, which include proteins like synaptotagmin like proteins (SLP-1).^138^ The SLP-1 protein itself is comprised of an N-terminal and a C-terminal.^139^ The role of SLP-1 is unclear in tumour progressions but has been found upregulated in prostate cancer cell lines^140^ and endometrial cancer,^141^ and its use has been suggested as a potential biomarker.

### E-Cadherin

Cadherins are the calcium-dependent (hence the name) molecules that are responsible for forming cell-to-cell adhering complexes,^142^ maintaining cellular structure by reinforcing cytoskeletal elements,^143^ resisting cellular damage, and also participating in cellular signalling pathways.^144^ Cadherins are of various types and have distinct functions but depending on their location, they are divided into E-cadherins (epithelial cells), N-cadherins (neurons), R-cadherin (retinal), VE-cadherin (vascular endothelial), K-cadherin (kidney), H-cadherin (heart), OB-cadherins (osteoblasts), M-cadherins (myotubule), LI-cadherin (liver-intestine) and, P-cadherins (placenta).^145^ Given the nature of tonsillar tumours, it can be argued that E-cadherins would be the most important in tumorigenic processes involving the tonsil, oral cavity or oropharynx. E-cadherins, given their adhesive function, are one of the most crucial tumour suppressive molecules and prevent the collection of neoplastic cells to detach from the epithelial membrane and metastasize into distant tissues.^146,147^ This exchange of information and molecules is referred to as epithelial-mesenchymal transition.^147^

### β-Catenin

Adhesion and transcription are two key cellular events that regulate carcinogenic transformations, and β-catenin is a protein known to be involved in both processes.^148–150^ Wnt/β-catenin signalling is a key pathway that regulates almost all cellular events (differentiation, apoptosis, renewal and proliferation). β-catenin is the single most critical component of the complex which needs to be stable to activate this pathway; thus, regulates essential cellular processes. Any deviation or instability can influence these processes, resulting in neoplastic consequences.^151^ Dysregulated genetic expressions of this protein have been associated with cancers of the colon,^152–154^ breast,^155–158^ ovaries^159–161^ and liver.^162–164^ Additionally, β-catenin’s linked to cardiac diseases like cardiomyopathy^165^ and congenital heart disorders^166^ and, behavioural issues like depression and stress.^167–169^

### LMP7 - Low Mass Protein-7

Low mass proteins 7 and 2 both have a protective effect against cancer antigens and are known to play an active role in immune surveillance via regulating the MHC-1 pathways.^170–172^ Genetic variations in the DNA sequence for LMP-7 have been associated with altered functional capability thus impacting the antigen processing mechanism and efficiency and is frequently associated with cancers of the colon,^173,174^ cervix,^175,176^ blood cells,^177^ and gastric tract.^178,179^ The association with cervical cancers highlights the connection with HPV infections and also a probable impact on the development of the tonsillar, oropharyngeal and other HPV infection-associated cancers.^179^ It has already been reported that polymorphism in these genes leads to defects in protein structure and functions eventually impacting the ability of the infected person to clear the infection.^176^ Deshpande et al,^176^ also elaborates that any genes or proteins involved with the antigen processing for HLA class 1 proteins may have an impact on malignant transformations of cells.

### E2

E2 proteins are critical to the replication and transcription processes of the papillomavirus. It is linked to numerous viral survival and progressing processes, as well as proteins involved in the viral life cycle.^180^ E2 proteins have been frequently linked to the carcinogenic pathways associated with persistent HPV infection.^181–183^ A recent systematic review provided evidence of the E2 protein of HPV-16 to be involved with cellular death, via apoptotic pathways; although, the mechanism was still not well understood.^184^ The role of these proteins is not well understood, a few studies^30,31^ have not reported any significance so far, but further research is required to establish its specific role in tonsillar cancers.

### E6 and E7

E6 and E7 are oncogenic viral proteins, which instigate cellular processes like angiogenesis,^185,186^ uncontrolled cell division,^187,188^ metastasis^189^ etc; thus, critical to oncogenesis. Evidence also shows that E6 and E7 prevents apoptosis by destroying apoptosis inducing factors, leading to the production and persistent HPV affected cells.^190,191^ Uninhibited E6/E7 expression mimics mutational activity of pRb and p53, resulting in an unstable genome, mutations, malignant changes and other cellular changes favourable for malignant transformation.^192^ The role of E6/E7 oncoproteins in HPV induced malignancies is well defined and thoroughly reported.^189^ Needless to say, these proteins play a critical role in HPV associated carcinogenesis, and are currently considered the gold-standard for diagnosing HPV-associated lesions of the head and neck.

## Limitations

As all research papers and studies, the current review also has some limitations. It is primarily a literature review, an attempt to collate all the evidence which could potentially result in clinical implications for HPV-associated tonsillar cancers. Another limitation is that all the proteins identified have been discussed in limited capacity with brief correlations with HPV infections and their role in cancer initiation and progress. Although, this does compile all the theoretical evidence, translational outcomes of this evidence are yet to be researched and implemented. Another limitation is the geographical distribution of the papers included, which was primarily based out of research from Europe, and there is currently a lack of data/research from lower socio-economic countries, where the burden of HPV associated cancers is much higher. Given that this review is based of secondary evidence as generated by studies carried out in different populations, using different methods of investigation and sample size invariances, there is a potential of bias as well.

## Conclusion

A clear understanding of the cellular interactions and protein expressions is critical whilst making clinical management decisions. The literature regarding the exact bimolecular mechanism of action for HPV-associated tonsillar cancers is inconclusive and lacks substantial evidence. This review provides a comprehensive overview of the proteins identified and the hypothesized pathogenesis, but further research targeting especially HPV infected cells and the related tumorigenesis process is recommended. The biomolecules and genetic processes involved in the malignant transformation of each cell type are unique and diagnostic as well as therapeutic decisions should not be made based on assumptions. Thus, further corroborating the need for focused bimolecular research for viral-induced carcinogenesis.

## Supplemental Material

Supplemental Material - Defining the Molecular Intricacies of Human Papillomavirus-Associated Tonsillar Carcinoma Supplemental Material for Defining the Molecular Intricacies of Human Papillomavirus-Associated Tonsillar Carcinoma by Sneha Sethi in Cancer Control
