# Supplementary material for: Defining the Molecular Intricacies of Human Papillomavirus-Associated Tonsillar Carcinoma
Source: Cancer Control. 2025 May 7;32:10732748241310932. doi: 10.1177/10732748241310932 (PMC12062609; doi:10.1177/10732748241310932)
Supplement: Supplemental Material - Defining the Molecular Intricacies of Human Papillomavirus-Associated Tonsillar Carcinoma [file sj-pdf-1-ccx-10.1177_10732748241310932.pdf]

Appendix 1: Logic Grid Search

30th July, 2023

Logic Grid: **Pubmed: 745**

HPV AND TONSILLAR CANCER

HPV	Tonsillar Cancer
HPV[Text Word] OR "papillomaviridae"[MeSH Terms] OR “human papillomavirus”[MeSH] OR “human papillomavirus”[Text Word] OR "human papillomavirus 16"[MeSH Terms] OR human papillomavirus 16 [Text Word] OR "human papillomavirus 18" [MeSH Terms] OR human papillomavirus 18 [Text Word] OR papillomavirus[Text Word]	("Tonsils"[Text Word] OR "tonsil*"[All Fields]) AND ("cancer"[Text Word] OR "carcinoma"[Text Word] OR "Malignant"[Text Word])

Logic Grid: **EMBASE: 543**

HPV	Tonsillar Cancer
('human papillomavirus type 16':ti,ab,kw OR 'human papillomavirus type 18':ti,ab,kw OR 'wart virus':ti,ab,kw OR 'oral human papillomavirus infection':ti,ab,kw OR 'hpv':ti,ab,kw)	‘tonsil’:ti,ab,kw OR 'tonsils':ti,ab,kw OR ‘tonsillar cancer' OR ‘tonsillar malignan*’ OR ‘tonsillar carcinoma’

Logic Grid **SCOPUS: 320**

HPV	Tonsillar cancer
TITLE-ABS-KEY (HPV) OR TITLE-ABS-KEY (papillomaviridae) OR TITLE-ABS-	TITLE-ABS-KEY (tonsil and cancer) OR TITLE-ABS-KEY (tonsil AND malignant) OR TITLE-

KEY (human papillomavirus) OR TITLE-ABS-KEY (human papillomavirus type 16) OR TITLE-ABS-KEY (human papillomavirus type 18)	ABS-KEY (tonsillar carcinoma)

Web of Science: 1126

HPV	Tonsillar cancer
(HPV OR human papillomavirus OR human papillomavirus type 16 OR human papillomavirus type 18 OR Oral HPV OR wart virus)	(‘tonsil’ OR 'tonsils' OR ‘tonsillar cancer' OR ‘tonsillar malignan*’ OR ‘tonsillar carcinoma’)
